# A challenging diagnosis of interstitial ectopic pregnancy confounded by sonographic signs of adenomyosis: a case report

**DOI:** 10.1093/jscr/rjag003

**Published:** 2026-01-25

**Authors:** Grace Sutherland, Sophie Putt, Kapilesh Balachandar, Mark Ruff, Sashi Siva

**Affiliations:** Department of Obstetrics and Gynaecology, Northern Beaches Hospital – Obstetrics and Gynaecology, 105 Frenchs Forest Rd, Frenchs Forest, NSW 2086, Australia; Department of Obstetrics and Gynaecology, Northern Beaches Hospital – Obstetrics and Gynaecology, 105 Frenchs Forest Rd, Frenchs Forest, NSW 2086, Australia; Northern Beaches Hospital – Beaches Advanced Gynaecological Surgery, 105 Frenchs Forest Road, Frenchs Forest NSW 2086, Australia; School of Medicine, Anderson Stuart Building, The University of Sydney, Camperdown NSW 2050, Australia; Northern Beaches Hospital – Beaches Advanced Gynaecological Surgery, 105 Frenchs Forest Road, Frenchs Forest NSW 2086, Australia; Sydney Ultrasound for Women, Northern Beaches Hospital, Suite 5, Level 6, 105 Frenchs Forest Rd, Frenchs Forest NSW 2086, Australia

**Keywords:** interstitial ectopic pregnancy, adenomyosis, early pregnancy, ultrasound diagnosis, laparoscopic wedge resection, fertility preservation

## Abstract

Interstitial ectopic pregnancies account for 1%–3% of ectopic pregnancies and carry a high risk of morbidity due to delayed diagnosis and potential catastrophic haemorrhage. Sonographic identification is challenging and may be further confounded by distorted pelvic anatomy, including adenomyosis, which can present with myometrial cysts or sub-endometrial microcysts that mimic a gestational sac.

We describe a 36-year-old woman presenting with early-pregnancy bleeding whose initial ultrasounds suggested a possible intrauterine pregnancy due to adenomyosis-related cystic changes. Serial serum human chorionic gonadotropin (hCG) levels rose sub-optimally, and specialist ultrasound ultimately confirmed a right interstitial ectopic pregnancy. She underwent successful laparoscopic uterine wedge resection and bilateral salpingectomy with the use of intramyometrial vasopressin and tranexamic acid to minimize blood loss. This case highlights the importance of vigilance in pregnancies of unknown location, particularly when adenomyosis distorts sonographic anatomy, and demonstrates the role of minimally invasive techniques and haemostatic adjuncts in reducing morbidity.

## Introduction

Ectopic pregnancies occur in 1%–2% of all pregnancies and remain a major cause of early pregnancy morbidity [1]. Risk factors include prior ectopic pregnancy [2], smoking, intrauterine device us,e, and distorted pelvic anatomy [3]. Interstitial ectopic pregnancies account for 1%–3% of all ectopic pregnancies and are associated with significant morbidity due to delayed diagnosis and catastrophic haemorrhage [4].

Ultrasound features used to diagnose interstitial pregnancies include an eccentrically located gestational sac, a thin surrounding myometrial mantle, the ‘interstitial line sign,’ and absence of communication between the sac and endometrial cavity [5, 6]. These features may be difficult to interpret in patients with distorted uterine anatomy.

Adenomyosis is characterized by ectopic endometrial glands and stroma within the myometrium and is associated with ultrasound findings such as myometrial cysts, sub-endometrial microcysts, echogenic islands, and subendometrial buds [7]. Sub-endometrial microcysts are particularly predictive of adenomyosis [8], and adenomyosis affects ~10% of women with subfertility [9]. These features may mimic early intrauterine pregnancy structures, confounding assessment of pregnancies of unknown location [10].

Laparoscopic cornual resection is the preferred surgical management for interstitial ectopic pregnancy, offering reduced hospital stay and comparable operative outcomes to laparotomy [11]. Haemostatic techniques such as intramyometrial vasopressin injection improve visualization and reduce blood loss [12, 13]. Purse-string suture techniques may further reduce bleeding risk during resection [14].

This case highlights diagnostic delay due to adenomyosis-related cystic changes mimicking early intrauterine pregnancy.

## Case presentation

A 36-year-old woman, gravida 2 para 1, presented at 9 + 1 weeks’ gestation with heavy vaginal bleeding. The pregnancy was spontaneously conceived during a downregulation cycle preceding in vitro fertilisation for secondary infertility. Her history included a prior term vaginal delivery, treated *Chlamydia trachomatis* infection, adenomyosis, and bilateral tubal occlusion on hystero-contrast salpingography.

Her initial serum hCG rose sub-optimally from 2240 to 3420 IU/L over 48 hours (53%). An ultrasound performed at a general radiology practice did not demonstrate an intrauterine or extrauterine pregnancy but described a 15 × 16 mm avascular cystic structure suggestive of a possible gestational sac. She was managed with serial hCG testing in view of the wanted pregnancy and equivocal imaging.

Three days later, she developed right iliac fossa pain. Her hCG had increased to 7562 IU/L and repeat ultrasound suggested a possible gestational sac and yolk sac. She remained stable and was discharged with close follow-up for pregnancy of unknown location.

Eight days after initial presentation, her hCG level was 9430 IU/L. A specialist obstetric and gynaecological ultrasound on Day 10 demonstrated severe adenomyosis but no intrauterine or extrauterine gestation. She re-presented at Day 17, with hCG rising to 18 424 IU/L.

**Figure 1:**
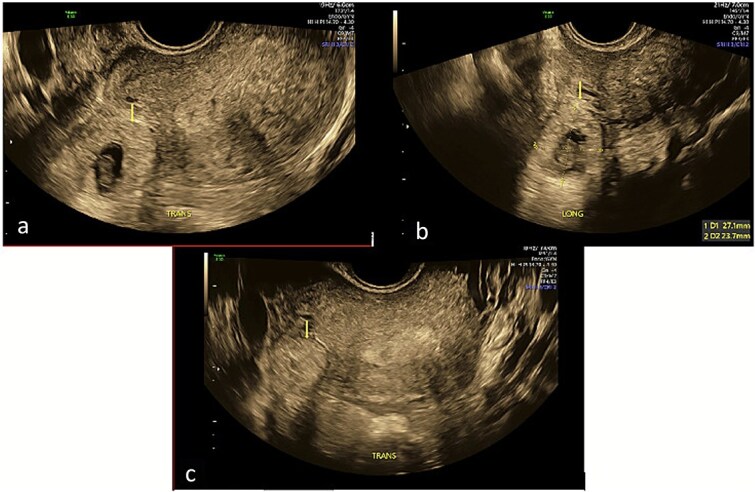
Transverse and longitudinal views of the uterus on trans-vaginal ultrasound demonstrating a right interstitial ectopic pregnancy with gestational sac and foetal pole. Image A demonstrates a decidual reaction around the gestational sac in a transverse plane through the uterus. Image B measures the gestational sac in the longitudinal plane. Image C demonstrates that the pregnancy does not communicate with the endometrial cavity in a transverse plane through the uterine fundus.

**Figure 2:**
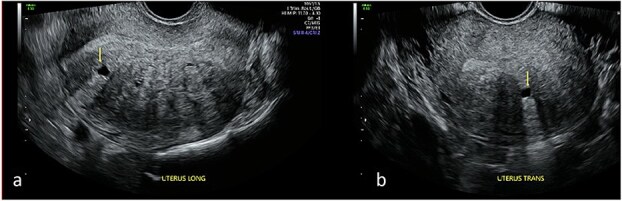
Transvaginal ultrasound of the uterus demonstrating adenomyotic spaces in the myometrium and sub-endometrial border in longitudinal (a) and transverse planes (b), with a notable absence of decidual reaction around the cystic spaces.

**Figure 3:**
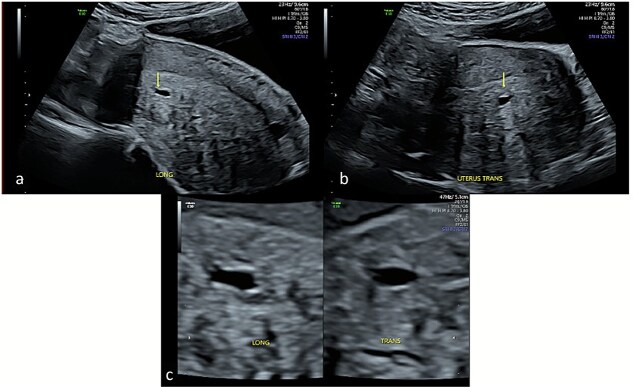
Transabdominal ultrasound of the uterus demonstrating cystic sub-endometrial spaces that were mistaken for a gestational sac in earlier ultrasound scans. The sub-endometrial space is demonstrated in longitudinal (a) and transverse planes (b) and under magnification (c) with a notable absence of decidual reaction around the cystic space.

Repeat specialist ultrasound (Fig. 1) identified a right interstitial ectopic pregnancy measuring 27 × 24 × 26 mm, with a decidual reaction, a foetal pole and absent cardiac activity. The gestational sac did not communicate with the endometrial cavity. Diffuse cystic adenomyosis was noted (Fig. 2). Retrospective review indicated that sub-endometrial microcysts had been mistaken for a gestational sac on earlier scans (Fig. 3).

**Figure 4:**
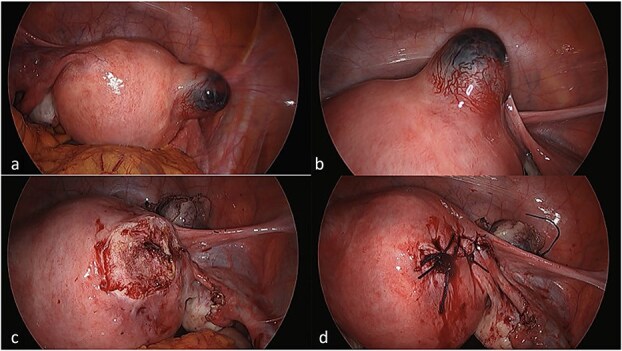
Laparoscopic views of the interstitial ectopic pregnancy. Part A and B demonstrate the pregnancy arising off the interstitial segment of the right fallopian tube at different magnifications, after entry into the peritoneal cavity. Part C is a progress image of the uterus after a unilateral salpingectomy and wedge resection. Part D is a progress image of the uterus after it was closed with a barbed v-lock suture.

She underwent laparoscopic uterine wedge resection and bilateral salpingectomy. On entering the peritoneal cavity, the unruptured ectopic pregnancy was clearly visible (Fig. 4a and b). Intramyometrial vasopressin (4 IU) and intravenous tranexamic acid (1 g) were administered. The interstitial pregnancy was excised using a Harmonic scalpel. The myometrial defect was closed with barbed suture and the serosa with interrupted polyglactin (Fig. 4c and d). Her recovery was uneventful, and she later conceived an intrauterine pregnancy with assisted reproduction.

## Discussion

This case demonstrates how adenomyosis can confound early pregnancy imaging, leading to delayed diagnosis of interstitial ectopic pregnancy. Myometrial cysts and sub-endometrial microcysts—common in adenomyosis—may resemble an early gestational sac, particularly in pregnancies of unknown location. Sub-endometrial microcysts are among the strongest ultrasonographic predictors of adenomyosis [8], and the prevalence is higher among women with subfertility [9]. In this case, adenomyosis-related cystic spaces contributed directly to misinterpretation and delayed recognition of the interstitial pregnancy.

Interstitial pregnancies may be difficult to diagnose even under optimal conditions. Key diagnostic features, including the interstitial line sign and absence of communication with the endometrial cavity, were clearly visualised only upon specialist ultrasound assessment (Fig. 1), underscoring the importance of high-resolution imaging and expertise.

In stable patients with unruptured interstitial ectopic pregnancies, laparoscopic management is preferred. A systematic review found equivalent operative outcomes but shorter length of stay compared with laparotomy [11]. Haemostatic adjuncts such as intramyometrial vasopressin are commonly used to reduce bleeding and improve operative visibility [12, 13]. More recently, purse-string techniques combining vasopressin injection with circumferential suturing have shown excellent haemostatic results [14]. These principles were reflected in this case, where intraoperative vasopressin contributed to minimal blood loss and an uncomplicated postoperative course.

## Conclusion

Adenomyosis may mimic early pregnancy structures on ultrasound and delay diagnosis of interstitial ectopic pregnancy. Specialist imaging and close surveillance of pregnancies of unknown location are essential. Laparoscopic management with haemostatic adjuncts such as vasopressin can be safely and effectively performed in stable patients.

## Learning Points

- Adenomyosis can mimic early intrauterine pregnancy, delaying recognition of interstitial ectopic pregnancy.
- Specialist ultrasound assessment is crucial when hCG rises abnormally and imaging is inconclusive.
- Laparoscopic cornual resection with haemostatic adjuncts can be performed safely in stable patients
